# Psychotropic dispensing in Greek adolescents and young adults: national trends and regional variation

**DOI:** 10.3389/fdsfr.2026.1900494

**Published:** 2026-08-25

**Authors:** Konstantinos Kassandros, Maria Samakouri, Maria Panagopoulou, Theodoros Constantinidis, Christos Kontogiorgis

**Affiliations:** 1 Laboratory of Hygiene and Environmental Protection, Department of Medicine, Democritus University of Thrace, Alexandroupolis, Greece; 2 Department of Psychiatry, Faculty of Medicine, Democritus University of Thrace, Alexandroupolis, Greece

**Keywords:** antidepressants, antipsychotics, benzodiazepines, Greece, pediatric, pharmacoepidemiology, psychotropics, young adults

## Abstract

**Purpose:**

The study quantified national trends in psychotropic medication dispensing among children and adolescents (0–17 years) and young adults (18–29 years) in Greece during 2020–2024 and characterized patterns by drug class, sex, diagnosis, and region.

**Methods:**

Repeated cross-sectional analyses used nationwide administrative e-prescription dispensing data (used as a proxy for medication exposure) aggregated by year, age group, and sex. Outcomes were total dispensed packs, dispensing prevalence per 1,000 inhabitants, and dispensing intensity (packs per patient). Pharmacologic classes followed Anatomical Therapeutic Chemical (ATC) groupings, while International Classification of Diseases, 10th Revision (ICD-10) macro-groups were constructed to link dispensing with psychiatric and selected G-chapter and related diagnostic categories.

**Results:**

Overall psychotropic dispensing prevalence increased from 64.5 to 71.8 per 1,000 inhabitants between 2020 and 2024. SSRI dispensing prevalence increased from 6.65 to 9.09 per 1,000, whereas hypnotic benzodiazepine prevalence declined from 1.01 to 0.94 per 1,000 and Z-drugs prevalence decreased from 0.20 to 0.15 per 1,000. Females had higher odds of antidepressant dispensing. Regional antidepressant dispensing prevalence was highest in the Ionian Islands (13.46 per 1,000), followed by Attica (11.93 per 1,000) and Western Greece (11.86 per 1,000).

**Conclusion:**

Psychotropic medication dispensing among Greek adolescents and young adults increased during 2020–2024, largely due to SSRIs, with stable dispensing intensity and marked heterogeneity by age, sex, diagnosis, and region. These findings support age-appropriate prescribing stewardship and targeted regional monitoring.

## 1 Introduction

The pharmacological treatment of mental health problems in adolescents and young adults has expanded over the last 15 years, with notable changes in the use of antidepressants, antipsychotics and benzodiazepines and Z-drugs. Globally, the dispensing-based psychotropic medication exposure increased during 2008–2019, with a wide range between countries and a stronger contribution of antidepressants, while at the same time, differences by income category and geographic area with higher usage and faster increases in higher income categories (Brauer et al., 2021). In European and other health systems, drug use studies document a steady rise in antidepressant administration in children/adolescents, with variations by age group and sex (Chua et al., 2024; Jack et al., 2020; Wesselhoeft et al., 2020).

In the United Kingdom (1998-2017), the US (2016–2022 with a more pronounced increase in the COVID-19 period), Italy (interrupted time series following the pandemic) and Nordic countries (prior to the pandemic) increasing trends in the use of antidepressants in children/adolescents were documented (Chua et al., 2024; Jack et al., 2020; Di Valerio et al., 2024; Gallinella et al., 2024; Rasmussen et al., 2025).

Antipsychotic use in the pediatric population remains heterogeneous but with clear signs of an increase over the last decade. In Germany (2011–2020) and in England (2000–2019), increasing trends in pediatric antipsychotic prescribing were reported, including a more pronounced increase in older adolescents and changes in prescribing patterns, mainly due to an increase in quetiapine (Dörks et al., 2023; Radojčić et al., 2023). Pan-European work has highlighted wide variation in pediatric antipsychotic use and frequent off-label prescribing (Kaguelidou et al., 2020; Varimo et al., 2020), while evidence on physiological and metabolic effects of antipsychotics in children and adolescents, continues to inform benefit-risk considerations (Rogdaki et al., 2024).

The use of benzodiazepines (BZD) and related hypnotics in young people presents additional concerns. Swedish registries (2006–2013) demonstrated prescribing of BZD in adolescents/young adults and off-label dispensing, with variations by age/sex (Sidorchuk et al., 2018) while a French cross-sectional study linked social deprivation with higher odds of dispensing benzodiazepines in children/adolescents (Driot et al., 2023). Irish evidence identified benzodiazepine prescribing patterns in children that require monitoring (O’Sullivan et al., 2015), while longer term analyses beyond Europe reported complex temporal trends in sedatives-hypnotics use across subclasses, including in young adults (Kwok et al., 2025).

In the Greek context, a recent national analysis of prescribing data (2016–2019) showed that pediatric psychotropic dispensing prevalence ranges approximately 3.1–6.5 per 1,000 with higher exposure in adolescents and around one in seven children were exposed to off-label use (with quetiapine as the most common), underscoring the need to systematically characterize national usage patterns (Pesiou et al., 2024).

## 2 Aim

This study investigates psychotropic medicine dispensing in children and adolescents (0-17 years) and young adults (18-29 years) in Greece, comparing patterns between key pharmacological classes (benzodiazepines, antidepressants, antipsychotics), mapping differences by sex/age/diagnosis/region and bridging a knowledge gap. The goal is to address an important evidence gap and provide population-based analysis from a Southern European setting that supports cross-country comparisons and informs more evidence-based prescribing and monitoring in these age groups.

## 3 Methods

### 3.1 Study design and data sources

A repeated cross-sectional analysis of central nervous system drug dispensing in Greece was performed for calendar years 2020–2024. The target population included children and adolescents aged 0–17 years and young adults aged 18–29 years. Dispensing e-prescription data came from the National Organization for the Provision of Health Services (EOPYY) and included Anatomical Therapeutic Chemical (ATC) classification and International Classification of Diseases, 10th Revision (ICD-10) diagnosis. Analyses were stratified by year, age group, sex, pharmacological class, ICD-10 macro-group, and region, as applicable. The population denominators were defined based on the 2021 Census of Hellenic Statistical Authority (ELSTAT) by age group, sex and regional unit. Regional denominators were used to align patient numerators with the corresponding population and avoid double counting in the national sum. Dispensing data were used as a proxy for medication exposure and do not necessarily reflect actual medication intake or adherence.

### 3.2 Dispensing metrics

Three types of measurements applied at national level and in age and sex stratifications were used. Total dispensing volume was defined as the number of dispensed packs in each layer (Versporten et al., 2018). Dispensing prevalence per 1,000 inhabitants was defined as the number of patients with at least one dispensed medication divided by the corresponding population, multiplied by 1,000:

$${\text{Dispensing}\;\text{prevalence}}_{y,a,s}=\frac{\sum {\text{patients}}_{y,a,s}}{\sum {\text{Population}}_{y,a,s}}\times 1000$$

Dispensing intensity was defined as the ratio of packs to patients with at least one dispensed medication:

$${\text{Dispensing}\;i\text{ntensity}}_{y,a,s}=\frac{\sum {\text{Packs}}_{y,a,s}}{\sum {\text{Patients}}_{y,a,s}}$$

where y is the year, a the age group and s the sex. Zero or incomplete denominators led to mapping as an empty result.

### 3.3 Drug classification (ATC) and ICD-10 grouping

Pharmacological classification was based on the ATC system. Harmonization of names and classification levels was performed, with cross-referencing against official ATC descriptions. For presentation purposes, functional classes were constructed to group medications with similar therapeutic mechanisms. To enhance clinical interpretability, pharmacological classes were defined using a predefined substance-level classification framework independent of ATC subclass structure, as the ATC system does not distinguish between first- and second-generation antipsychotics. Full mappings of ATC codes for pharmacological classes are provided in Supplementary Table S1.

Cumulative counting of patients across pharmacological classes was not performed in order to avoid double counting of individuals receiving medications from multiple categories. Dispensed packs were considered additive and were summarized by class.

ICD-10 macro-groups were constructed based on predefined code ranges within the F chapter (mental and behavioural disorders) and selected categories from the G chapter with neurological relevance. Groupings were defined *a priori* according to the ICD-10 hierarchical structure, and ambiguous or infrequently used codes were manually reviewed and assigned based on their primary diagnostic category. Full ICD-10 group mappings are provided in Supplementary Table S2. For the heatmap analysis, diagnosis-specific dispensing prevalence was calculated as the number of patients with at least one dispensed medication in each pharmacological class and ICD-10 macro-group divided by the corresponding population, multiplied by 1,000. Dispensing intensity was calculated as the number of dispensed packs divided by the number of patients with at least one dispensed medication in the corresponding cell.

### 3.4 Regional harmonization and analysis

Regional analyses were conducted using the Nomenclature d' Unités Territoriales Statistiques (NUTS-2) administrative regions (13 regions), corresponding to the official statistical regional classification of Greece (Antoniou et al., 2023; Bushnell et al., 2023). NUTS-2 were defined as follows: Eastern Macedonia & Thrace, Central Macedonia, Western Macedonia, Epirus, Thessaly, Ionian Islands, Western Greece, Central Greece, Attica, Peloponnese, North Aegean, South Aegean, and Crete. For each year, metrics per regional unit and summary statistics of dispersion were calculated for dispensing prevalence per 1,000 and the dispensing intensity.

### 3.5 Comparative statistics

Comparisons between demographic groups were based on odds ratios (ORs) and 95% confidence intervals (Rasmussen et al., 2025; Zito et al., 2020; Gangapersad et al., 2024). OR analyses were performed only for comparisons between age groups and sexes. Temporal trends were described using annual values. No formal inferential tests of temporal trend were performed. For the pooled 2020–2024 comparisons, annual counts of individuals with at least one dispensed medication and the corresponding population denominators were summed across the study years within each comparison group before ORs and 95% confidence intervals were calculated. The pooled estimates therefore represent aggregated annual observations rather than unique individuals followed longitudinally. For age comparisons, the 0-17/18–29 groups were defined, stratified by year and sex:

$$OR=\frac{\frac{c}{N_{c}-c}}{\frac{a}{N_{a}-a}}=\frac{c\;\left(N_{a}-a\right)}{a\;\left(N_{c}-c\right)},$$

where: a = number of patients aged 18–29 years, $N_{a}$ = total population aged 18–29 years, c = number of patients aged 0–17 years, Nc = total population aged 0–17 years. For sex comparisons, Female/Male, by year and age group, OR was defined:

$$OR=\frac{\frac{a_{F}}{N_{F}-a_{F}}}{\frac{a_{M}}{N_{M}-a_{M}}}.$$

where: $a_{F}$ = number of female patients, $N_{F}$ = total female population, $a_{M}$ = number of male patients, $N_{M}$ = total male population, 95% confidence intervals were calculated using standard error of the log(OR):

$$SE=\sqrt{\frac{1}{a}+\frac{1}{b}+\frac{1}{c}+\frac{1}{d}},$$

where: $b=N_{a}-a\;\left(non-cases\;in\;the\;18-29\;group\right)$ , $d=N_{c}-c$ (non-cases in the 0–17 group).

## 4 Results

In this study, national psychotropic dispensing data among children/adolescents and young adults in Greece during 2020–2024 are reported. Overall dispensing and annual changes are presented first, followed by differences by age group and sex. Diagnostic distributions and regional variation are then reported to contextualize dispensing across clinical and geographic settings.

### 4.1 Demographics

The study population included patients aged 0–17 and 18–29 years across the period 2020–2024. In all years, the majority of patients were in the 18–29 age group, with males consistently representing the largest subgroup.

In the 0–17 age group, total patients increased from 7,142 in 2020 to 9,444 in 2022, followed by a decrease to 9,061 in 2024. Females exceeded males from 2021 onwards.

In the 18–29 age group, total patients increased from 74,059 in 2020 to 89,208 in 2024. Males remained the predominant subgroup, although female patient numbers increased steadily over time. Overall, the annual number of individuals with at least one dispensed psychotropic medication increased from 81,201 (64.5 per 1,000) in 2020 to 98,269 (71.8 per 1,000) in 2024. The summary is demonstrated in Table 1.

**TABLE 1:** Annual number of individuals with at least one dispensed psychotropic medication, by age group and sex, Greece, 2020–2024. Values represent the annual number of unique patients with at least one dispensed psychotropic medication, stratified by age group and sex. Age groups were defined as 0–17 and 18–29 years. Totals correspond to the sum of male and female patients within each age group and year.

Year	Age group, years	Male, n	Female, n	Total, n
2020	0–17	3,649	3,493	7,142
2020	18–29	42,343	31,716	74,059
2021	0–17	4,209	4,515	8,724
2021	18–29	46,273	36,879	83,152
2022	0–17	4,310	5,134	9,444
2022	18–29	47,237	38,618	85,855
2023	0–17	4,098	4,887	8,985
2023	18–29	46,864	40,221	87,085
2024	0–17	4,244	4,817	9,061
2024	18–29	47,438	41,770	89,208

### 4.2 Overall dispensing

**FIGURE 1:**
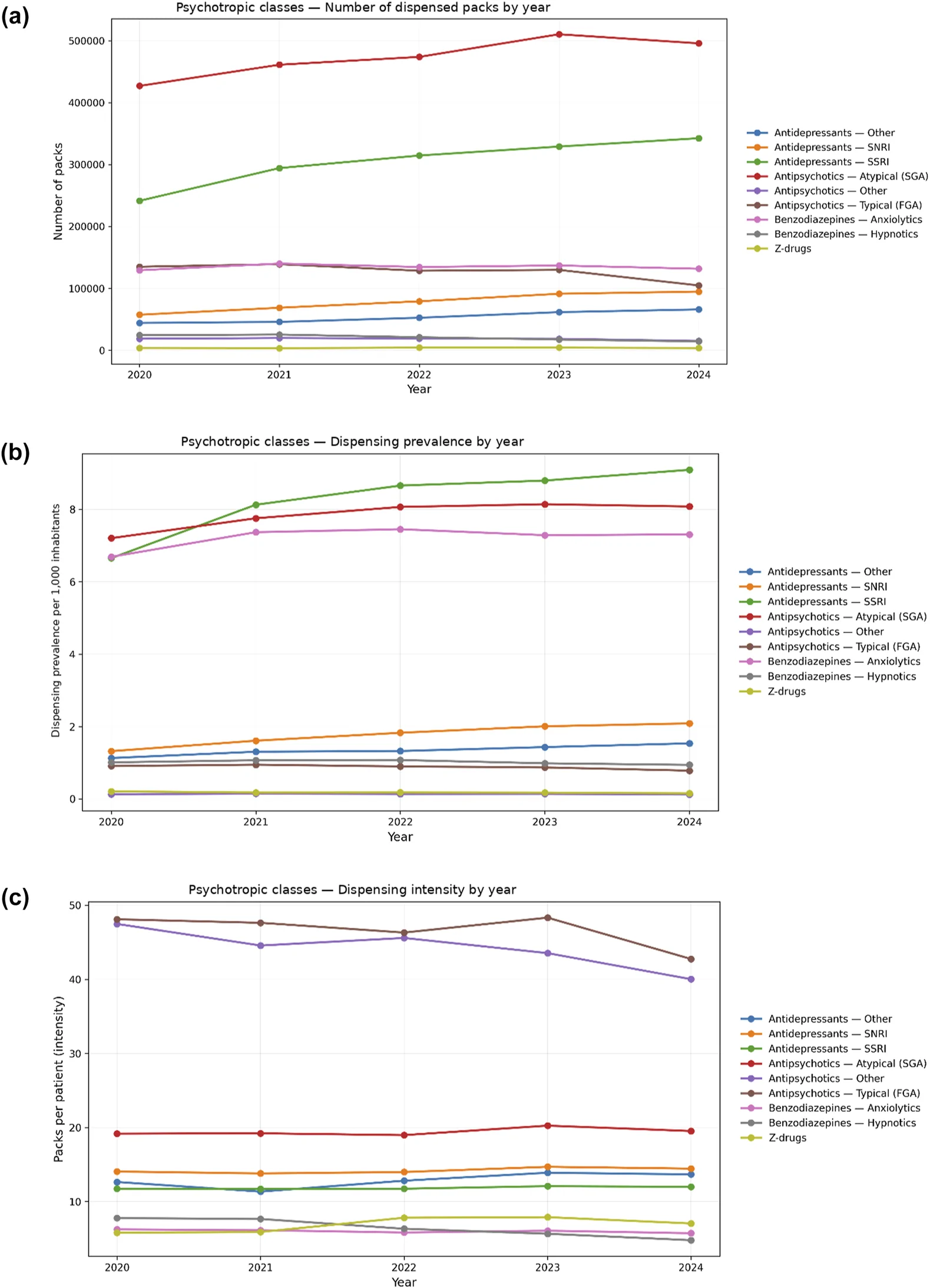
National trends in psychotropic dispensing in Greece (2020–2024) among children/adolescents (0–17 years) and young adults (18–29 years). **(a)** Annual total volume expressed as number of dispensed packs; **(b)** population-adjusted dispensing prevalence per 1,000 inhabitants, calculated using the number of patients with ≥1 dispensed prescription divided by the corresponding population; **(c)** dispensing intensity (packs per patient), calculated as total dispensed packs divided by the number of individuals with at least one dispensed medication. All measures are based on administrative e-prescription dispensing data and reflect medication exposure rather than confirmed use.

The total dispensing volume increased from 1,079,768 packs in 2020 to 1,298,005 in 2023 and declined mildly in 2024 to 1,266,823. The overall dispensing prevalence increased from 64.5 to 71.8 patients per 1,000 inhabitants. Dispensing intensity increased slightly in 2020–2022 from 13.30 to 13.38 packs per patient, increased in 2023 (13.51 packs per patient), and stabilized lower in 2024 (12.89 packs per patient). Class-specific dispensing prevalence and dispensing intensity in 2020 and 2024 are summarized in Table 2, while annual trends across volume, prevalence, and intensity are presented in Figure 1.

**TABLE 2:** Class-specific psychotropic dispensing prevalence and dispensing intensity in 2020 and 2024. Dispensing prevalence is expressed per 1,000 inhabitants and dispensing intensity as packs per patient. BZD-ANX, anxiolytic benzodiazepines; BZD-HYP, hypnotic benzodiazepines; SSRI, selective serotonin reuptake inhibitors; SNRI, serotonin–norepinephrine reuptake inhibitors; SGA, second-generation antipsychotics; FGA, first-generation antipsychotics.

Class	Prevalence 2020, per 1,000 inhabitants	Prevalence 2024, per 1,000 inhabitants	Intensity 2020, packs/patient	Intensity 2024, packs/patient
BZD-ANX	6.69	7.31	6.24	5.73
BZD-HYP	1.01	0.94	7.77	4.78
Z-drugs	0.20	0.15	5.79	7.04
SSRIs	6.65	9.09	11.73	11.98
SNRIs	1.32	2.08	14.05	14.45
SGAs	7.20	8.08	19.16	19.52
FGAs	0.91	0.78	48.09	42.74

In benzodiazepine receptor agonists (BZRA), anxiolytic benzodiazepines (BZD-ANX) volume remained broadly stable increasing from 129,143 packs in 2020 to 131,503 packs in 2024. In contrast, hypnotic benzodiazepines (BZD-HYP) volume declined from 24,199 to 14,096 packs, while Z-drugs showed marginal decline in volume from 3,660 to 3,367 packs. In antidepressants (AD), total packs increased from 342,600 in 2020 to 516,662 in 2023, with a mild decline in 2024 (502,755). Selective serotonin reuptake inhibitors (SSRIs) volume increased from 241,323 to 342,340 packs between 2020 and 2024, while serotonin–norepinephrine reuptake inhibitors (SNRIs) volume increased from 57,258 to 94,615 packs.

In the antipsychotics (AP) class, second-generation antipsychotics (SGAs) volume increased from 426,894 packs in 2020 to 495,605 packs in 2024, whereas first-generation antipsychotics (FGAs) declined in volume (134,760 to 104,573 packs).

### 4.3 Age- and sex-stratified odds ratio analysis

**FIGURE 2:**
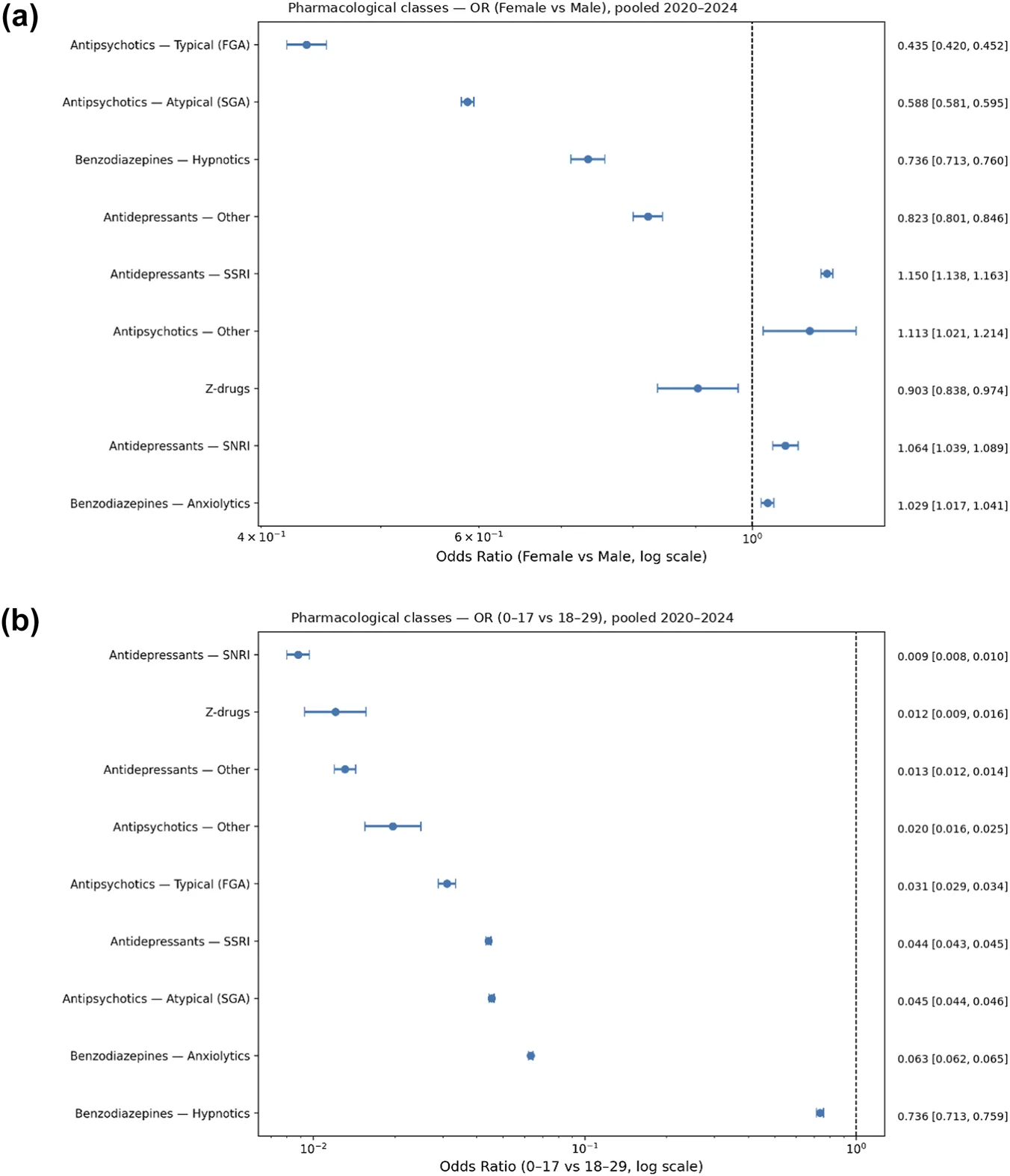
Forest plot of odds ratios (ORs) for psychotropic medication dispensing by sex and age group in Greece, 2020–2024. **(a)** Sex-specific ORs comparing females with males. **(b)** Age-specific ORs comparing children/adolescents aged 0–17 years with young adults aged 18–29 years. ORs with 95% confidence intervals are displayed on a logarithmic scale. Pooled estimates were calculated from annual dispensing-recipient counts and the corresponding population denominators summed across the study years. Values <1 indicate lower odds in the numerator group, and values >1 indicate higher odds. Estimates are calculated from administrative e-prescription dispensing data and reflect dispensing-based exposure.

For sex (female vs. male), ORs above 1.00 indicate higher odds in females, whereas ORs below 1.00 indicate lower odds in females (that is, relatively higher odds in males). Females had higher odds of SSRIs dispensing (OR = 1.150, 95% CI 1.138–1.163, p < 0.001), SNRIs (OR = 1.064, 95% CI 1.039–1.089, p < 0.001), other antipsychotics (OR = 1.113, 95% CI 1.021–1.214, p = 0.015), and benzodiazepine anxiolytics (OR = 1.029, 95% CI 1.017–1.041, p < 0.001). In contrast, females had lower odds for second-generation antipsychotics (SGA) dispensing (OR = 0.588, 95% CI 0.581–0.595, p < 0.001), first-generation antipsychotics (FGA) (OR = 0.435, 95% CI 0.420–0.452, p < 0.001), benzodiazepine hypnotics (OR = 0.736, 95% CI 0.713–0.760, p < 0.001), Z-drugs (OR = 0.903, 95% CI 0.838–0.974, p = 0.008), and other antidepressants (OR = 0.823, 95% CI 0.801–0.846, p < 0.001). This pattern reflects class-specific differences in dispensing by sex rather than a uniform direction of effect across all psychotropic categories (Figure 2).

Across age groups (0–17 vs. 18–29 years), an odds ratio (OR) below 1.00 indicates lower odds of having at least one dispensed medication in the 0–17 group relative to young adults. In the pooled 2020–2024 comparisons, lower odds were consistently observed in the 0–17 group across all classes, with the most pronounced differences for SNRIs (OR = 0.009, 95% CI 0.008–0.010, p < 0.001), Z-drugs (OR = 0.012, 95% CI 0.009–0.016, p < 0.001), other antidepressants (OR = 0.013, 95% CI 0.012–0.014, p < 0.001), and other antipsychotics (OR = 0.020, 95% CI 0.016–0.025, p < 0.001). Smaller but still substantial differences were observed for first-generation antipsychotics (FGA) (OR = 0.031, 95% CI 0.029–0.034, p < 0.001), SSRIs (OR = 0.044, 95% CI 0.043–0.045, p < 0.001), second-generation antipsychotics (SGA) (OR = 0.045, 95% CI 0.044–0.046, p < 0.001), and benzodiazepine anxiolytics (OR = 0.063, 95% CI 0.062–0.065, p < 0.001). Benzodiazepine hypnotics showed comparatively smaller differences between age groups, although odds remained lower in the 0–17 group (OR = 0.736, 95% CI 0.713–0.759, p < 0.001).

### 4.4 Heatmap analysis

In the 2020–2024 cumulative maps, the highest diagnosis-specific dispensing prevalence values were observed for SSRIs in Anxiety disorders (ANX; 5.06 per 1,000 inhabitants), followed by BZD-ANX in ANX (4.25 per 1,000) and SGA F20–F29 diagnostic group (PSY; 3.91 per 1,000).

**FIGURE 3:**
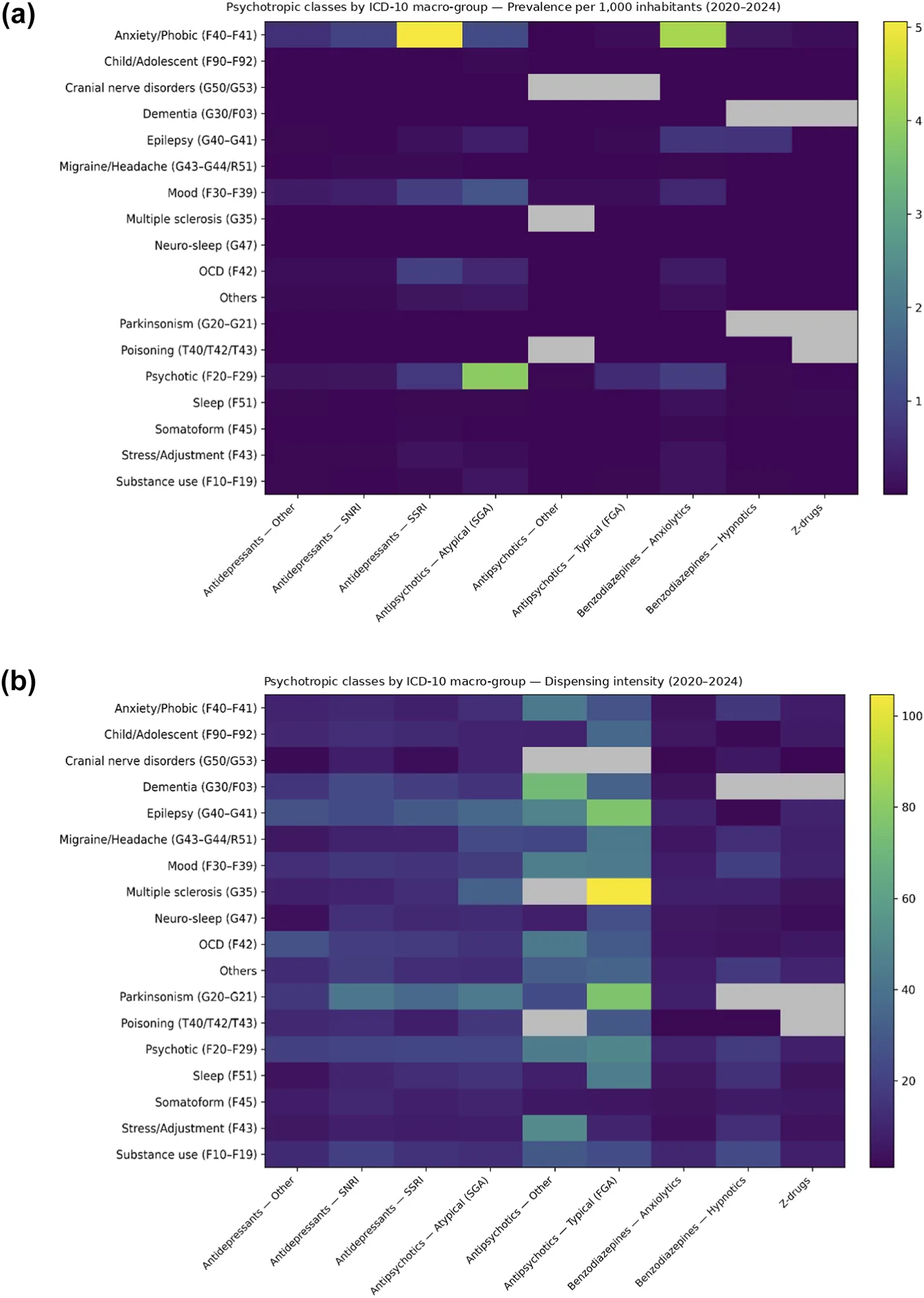
Heatmap representation of psychotropic dispensing patterns by ICD-10 macro-group and ATC pharmacological class in Greece (2020–2024). **(a)** Population-adjusted dispensing prevalence per 1,000 inhabitants; **(b)** dispensing intensity, defined as the number of dispensed packs per patient. ICD-10 macro-groups (rows) include psychiatric (F-chapter) and selected G-chapter and related diagnostic categories, while columns correspond to aggregated ATC drug classes. Each cell represents the pooled 2020–2024 estimate across the study period. Color gradients reflect increasing magnitude (lighter colors indicate higher values), and Gray cells indicate missing or non-applicable data. All measures are based on administrative e-prescription dispensing data and represent medication exposure rather than confirmed use.

The highest dispensing intensity values were observed for FGA in multiple sclerosis (MS; 104.67 packs per patient), parkinsonism and Parkinson’s disease–related disorders (PRK; 77.01) and EPI (76.84). The complete distribution across pharmacological classes and ICD-10 macro-groups is presented in Figure 3. These cell-level intensity estimates describe the number of packs dispensed per recipient and should not be interpreted as evidence of greater clinical need or treatment appropriateness.

### 4.5 Regional variation

Across 2020–2024, the national mean annual BZRA dispensing prevalence was 8.41 patients per 1,000 inhabitants. The highest rates were observed in the Ionian Islands (9.52 per 1,000), the Peloponnese (9.25 per 1,000), and Eastern Macedonia–Thrace (9.03 per 1,000), while the lowest were observed in the South Aegean (7.16 per 1,000), the North Aegean (7.41 per 1,000), and Central Greece (7.45 per 1,000).

For ADs, the population-weighted national mean annual dispensing prevalence was 11.38 per 1,000 inhabitants. The highest rates were observed in the Ionian Islands (13.46 per 1,000), Attica (11.93 per 1,000) and Western Greece (11.86 per 1,000), while the lowest were observed in the South Aegean (8.04 per 1,000), the North Aegean (9.68 per 1,000), and Central Greece (9.93 per 1,000).

For APs, the national mean annual dispensing prevalence was 8.86 per 1,000 inhabitants. The highest rates were observed in Western Greece (10.39 per 1,000), Crete (10.27 per 1,000), and the Peloponnese (9.89 per 1,000), while the lowest were observed in Epirus (6.73 per 1,000), the South Aegean (6.90 per 1,000), and Western Macedonia (7.05 per 1,000).

## 5 Discussion

From 2020 to 2024 total psychotropic dispensing prevalence increased among Greek children/adolescents and young adults, while dispensing intensity remained broadly stable. The concurrent increase in dispensing prevalence and the number of patients with at least one dispensed medication, without a corresponding increase in packs dispensed per patient, indicates that the rise in total dispensing volume was associated mainly with an increase in the number of recipients. The direction of change is consistent with findings from Ireland, where psychotropic medication use among young people increased from 6.41 to 8.46 per 1,000 between 2017 and 2021 (Parkin et al., 2025) and from Canada, where antidepressant use among young people was increased by 67.6% between 2014 and 2020 (Kitchen et al., 2025). However, direct numerical comparisons are limited by differences in age ranges, medication scope, healthcare organization, and observation periods.

Among the ages 0–17 years, dispensing prevalence increased through 2023 and declined slightly in 2024, while dispensing intensity decreased. Earlier BZD-specific registry studies provided historical context for changes in psychotropic dispensing among younger populations. In Sweden, benzodiazepine prescription prevalence among individuals aged 0–24 years increased from 0.81% to 0.99% between 2006 and 2013 (Sidorchuk et al., 2018), whereas in Ireland it decreased from 8.56 to 5.33 per 1,000 among children younger than 15 years between 2002 and 2011 (O’Sullivan et al., 2015). More recent evidence indicates that psychotropic prescribing patterns have continued to change. In France, the monthly psychotropic prescription rate among children and adolescents increased from 9.9 per 1,000 in January 2016 to 16.1 per 1,000 in May 2022, with particularly marked increases in anxiolytic, hypnotic and sedative, and antidepressant prescribing after the onset of the pandemic (Valtuille et al., 2024). An interrupted time-series analysis also identified increased trends in antidepressant and anxiolytic dispensing among adolescents in Norway and Sweden after March 2020, although corresponding changes were not observed in Italy (Pedersen et al., 2024).

Within benzodiazepine receptor agonists, dispensing prevalence increased modestly for anxiolytic benzodiazepines, whereas it declined for hypnotic benzodiazepines and Z-drugs. Dispensing intensity decreased for both benzodiazepine subclasses but increased for Z-drugs, indicating that population-level prevalence and dispensing volume per recipient may change in different directions. In Hong Kong, annual BZRA prescription prevalence increased from 1.88 to 5.69 per 1,000 between 2000 and 2020, while Z-drug prescribing among children declined over the same period (AAPC = −4.65%, 95% CI −9.94% to −1.06%) (Kwok et al., 2025) While intensity peaks in individual molecules or groups, previous Scandinavian evidence has emphasized long-term co-administration patterns, supporting the importance of drug-indication monitoring (Sidorchuk et al., 2018).

Antidepressant dispensing increased substantially, driven primarily by SSRIs, while dispensing intensity remained mostly stable. This indicates that the increase was associated mainly with a greater number of recipients rather than with greater dispensing volume per patient. The direction of change is consistent with European adolescent patterns reported during the pandemic period, including the increase recorded in the region of Emilia-Romagna, Italy and with national patterns in the UK (Di Valerio et al., 2024; Jack et al., 2020). SNRIs and other antidepressants also increased, indicating that the change was not restricted exclusively to SSRIs (Fassmer et al., 2025; Gómez-Lumbreras et al., 2021). The diagnosis-specific concentration of SSRI and SNRI dispensing in anxiety-related groups can be interpreted in the context of the 2023 World Federation of Societies of Biological Psychiatry guideline, which identifies SSRIs and SNRIs as first-line pharmacological options for anxiety disorders and includes recommendations addressing children and adolescents (Bandelow et al., 2023). The 2020 Royal Australian and New Zealand College of Psychiatrists guideline similarly places antidepressants within structured, diagnosis-specific treatment pathways for mood disorders, although the present aggregated dispensing data cannot determine guideline concordance or treatment appropriateness (Malhi et al., 2021).

When comparing 0-17 to 18–29 years, all psychotropic classes showed lower odds of receiving a dispensed medication in the pediatric group. This indicates that dispensing was concentrated primarily among young adults, but it does not imply lower clinical need or provide information on treatment appropriateness. Similar age-related differences in antidepressant and antipsychotic prescribing have been reported in other pediatric and young-adult populations (Radojčić et al., 2023; Wijlaars et al., 2012). Sex specific heterogeneity, for example, higher odds for sertraline and duloxetine in females but lower for escitalopram and venlafaxine, is consistent with sex diverse adolescent antidepressant series across Europe (Di Valerio et al., 2024; Fassmer et al., 2025).

For SGAs, dispensing prevalence increased, whereas FGAs prevalence declined, indicating a shift in the composition of antipsychotic dispensing. This pattern is consistent with increases in second-generation antipsychotic prescribing reported in northern and central European populations (Dörks et al., 2023; Varimo et al., 2020). The decline in FGA dispensing was directionally consistent with findings from the Italian ARITMO study noting differences in eras and age structures (Kaguelidou et al., 2020). The dispensing intensity observed for AP-OTH remained high, compatible with maintenance-oriented profiles and molecule specific burdens described (Rogdaki et al., 2024).

Regional comparisons demonstrated heterogeneity in population-adjusted dispensing prevalence across BZRAs, ADs, and APs. The regions with the highest prevalence were not identical across the three medication groups, suggesting that regional variation did not reflect a single uniform geographical pattern. Geographic variation in pediatric psychotropic medication dispensing has also been reported in Ontario, where differences were associated with regional and sociodemographic characteristics (Antoniou et al., 2023).

Within PSY, the diagnostic heatmaps showed a concentration of SGA mirroring the preference patterns reported in UK and Germany cohorts (Dörks et al., 2023; Radojčić et al., 2023). Antipsychotic dispensing across non organic and organic sleep disorders (SLP-F and SLP-G) categories is consistent with primary care observations of off-label antipsychotic use, particularly for sedative purposes, and aligns with meta-analytic evidence on their hypnotic effects (Klau et al., 2024; Lin et al., 2023). In OCD and related conditions, the observed use of antipsychotic classes further supports prescribing beyond their core primary indications, as previously described (Radojčić et al., 2023). Dispensing intensity within PSY remained highest for antipsychotic classes, exceeding 40 packs per patient in specific subgroups, consistent with maintenance-oriented treatment patterns reported in national and international series (Dörks et al., 2023).

In CHILD, the co-occurrence of antipsychotic and antidepressant classes across heatmaps aligns with first-line treatment drivers reported in UK primary care data, particularly for neurodevelopmental and mood-related conditions, and with broader shifts in prescribing patterns observed in Northern European settings, including increased use among adolescent females (Dörks et al., 2023; Radojčić et al., 2023; Varimo et al., 2020).

Limitations of the study include annual data, which reduces the ability to detect seasonality and short-term changes. Mapping at the ATC classification system without product, strength, and administration form information does not allow for the calculation of standardized consumption metrics such as defined daily doses (DDDs) per 1,000 inhabitants per day. Age stratification was rough (0–17 and 18–29), limiting the possibility of detailed investigation within the pediatric range. As the analysis is based on dispensing data, actual medication use, adherence, and consumption cannot be directly inferred. Cell-level dispensing intensity may be unstable when based on small numbers of recipients. Finally, the use of exclusively electronic prescriptions carries a risk of under recording non-electronic or out-of-system prescriptions, while the absence of a link with clinical severity, adverse reactions and socioeconomic indicators limits the interpretation of differences by region and sex.

## 6 Conclusion

From 2020 to 2024, psychotropic dispensing prevalence in Greek children/adolescents and young adults increased, while dispensing intensity was largely stable. This suggests that more individuals received psychotropic medications over time, without a proportional increase in the amount dispensed per patient. Growth was driven by SSRIs/SNRIs and SGAs, with declines in FGAs, persistent BZD-anxiolytic dispensing, and BZRA dispensing associated with epilepsy diagnoses. Children/adolescents 0–17 had markedly lower odds of having at least one dispensed medication across classes. Sex effects were drug-specific, regional and diagnostic heatmaps showed concentrated dispensing patterns across specific subgroups. These patterns support indication-focused stewardship and structured review/deprescribing, with future analyses using higher-frequency data and outcome linkages.

## Data Availability

The data analyzed in this study is subject to the following licenses/restrictions: The datasets analyzed in this study are not publicly available because they derive from national administrative e-prescription dispensing aggregates provided under institutional permission. Access is restricted by applicable legal, ethical, and institutional requirements. Requests to access the data should be directed to the relevant data holder and are subject to approval by the competent authorities. Requests to access these datasets should be directed to IDIKA S.A./Electronic Governance of Social Security S.A., dpo@idika.gr.

## Glossary

AD: antidepressants
AD-OTH: other antidepressants
ANX: anxiety/phobic diagnostic group, F40–F41
AP: antipsychotics
AP-OTH: other antipsychotics
ATC: Anatomical Therapeutic Chemical classification
BZD: benzodiazepines
BZD-ANX: anxiolytic benzodiazepines, N05BA
BZD-HYP: hypnotic benzodiazepines, N05CD
BZRA: benzodiazepine receptor agonists, including benzodiazepines and Z-drugs
CHILD: behavioural and emotional disorders with onset usually occurring in childhood and adolescence, F90–F92
CI: confidence interval
COVID-19: coronavirus disease 2019
DDD: defined daily dose
EAT: eating disorders, F50
ELSTAT: Hellenic Statistical Authority
EOPYY: National Organization for the Provision of Health Services
EPI: epilepsy, G40–G41
FGA: first-generation antipsychotics
HDG: headache and migraine diagnostic group, G43–G44 and R51
ICD-10: International Classification of Diseases, 10th Revision
IQR: interquartile range
MAOI: monoamine oxidase inhibitor
MOOD: mood or affective diagnostic group, F30–F39
MS: multiple sclerosis, G35
NaSSA: noradrenergic and specific serotonergic antidepressant
NDRI: norepinephrine-dopamine reuptake inhibitor
NUTS-2: Nomenclature of Territorial Units for Statistics, level 2
OCD: obsessive-compulsive disorder, F42
OR: odds ratio
OTHER-F: other F-chapter diagnostic categories
OTH-NEU: other selected G-chapter categories
p: p-value
PERS: personality disorders, F60–F69
POIS: poisoning-related categories, T40, T42, and T43
PRK: parkinsonism and Parkinson’s disease-related disorders, G20–G21
PSY: F20–F29 diagnostic group
SE: standard error
SGA: second-generation antipsychotics
SLP-F: non-organic sleep disorders, F51
SLP-G: organic sleep disorders, G47
SMS: serotonin modulators and stimulators
SNRI: serotonin-norepinephrine reuptake inhibitor
SOM: somatoform disorders, F45
SSRI: selective serotonin reuptake inhibitor
STR: stress-related and adjustment disorders, F43
SUB: substance use disorders, F10–F19
TCA: tricyclic antidepressant
ZDR: Z-drugs, N05CF
Z-drugs: non-benzodiazepine hypnotics, including zolpidem and zopiclone
