# Supplementary material for: Psychotropic dispensing in Greek adolescents and young adults: national trends and regional variation
Source: Front Drug Saf Regul. 2026 Aug 25;6:1900494. doi: 10.3389/fdsfr.2026.1900494 (PMC13550184; doi:10.3389/fdsfr.2026.1900494)
Supplement: Supplementary file 1 [file Table1.docx]

Supplementary Table S1: ATC pharmacological class mapping.

| **Category** | **Abbrev.** | **Class** | **ATC mapping** | **Molecules** |
| --- | --- | --- | --- | --- |
| **Antidepressants (AD)** |  |  |  |  |
|  | SSRIs | Antidepressants — SSRI | N06AB* | sertraline, escitalopram, fluoxetine, paroxetine, citalopram, fluvoxamine |
|  | SNRIs | Antidepressants — SNRI | N06AX16, N06AX21 | venlafaxine, duloxetine |
|  | AD-OTH | Antidepressants — Other | N06A* excluding N06AB*, N06AF*, N06AG*, N06AX16, N06AX21 | mirtazapine, bupropion, trazodone, vortioxetine, agomelatine; TCAs: clomipramine, amitriptyline, maprotiline, doxepin |
| **Antipsychotics (AP)** |  |  |  |  |
|  | SGA | Second-generation antipsychotics (SGA) | N05AE*, N05AH*, N05AX*, N05AL05 | risperidone, quetiapine, olanzapine, aripiprazole, paliperidone, amisulpride, clozapine, cariprazine, lurasidone, ziprasidone, asenapine, sertindole |
|  | FGA | First-generation antipsychotics (FGA) | N05AA*, N05AB*, N05AC*, N05AD*, N05AF*, N05AG*, N05AL01 | haloperidol, levomepromazine, chlorpromazine, trifluoperazine, zuclopenthixol, pimozide, pipamperone, sulpiride |
|  | AP-OTH | Other antipsychotics | Residual N05A agents not consistently classifiable into FGA or SGA based on pharmacological profile | — |
| **BZRA (benzodiazepine receptor agonists)** |  |  |  |  |
|  | BZD-ANX | Benzodiazepines — Anxiolytics | N05BA* | alprazolam, lorazepam, diazepam, bromazepam, clorazepate, chlordiazepoxide, clobazam |
|  | BZD-HYP | Benzodiazepines — Hypnotics | N05CD* | flunitrazepam, midazolam |
|  | ZDR | Z-drugs (non-benzodiazepine hypnotics) | N05CF* | zolpidem, zopiclone |

Supplementary Table S2 : ICD-10 diagnostic grouping framework.

| **Group** | **Abbrev.** | **Subgroup** | **ICD-10 codes** |
| --- | --- | --- | --- |
| F-chapter diagnostic categories | F20–F29 | F20–F29 diagnostic group | F20–F29 |
|  | MOOD | Mood (affective) disorders | F30–F39 |
|  | ANX | Anxiety/phobic disorders | F40–F41 |
|  | OCD | Obsessive–compulsive disorder | F42 |
|  | STR | Stress-related and adjustment disorders | F43 |
|  | SOM | Somatoform disorders | F45 |
|  | EAT | Eating disorders | F50 |
|  | SLP-F | Non-organic sleep disorders | F51 |
|  | PERS | Personality disorders | F60–F69 |
|  | SUB | Substance use disorders | F10–F19 |
|  | CHILD | Behavioural and emotional disorders with onset usually occurring in childhood and adolescence | F90–F92 |
| Selected G-chapter and related diagnostic categories | EPI | Epilepsy | G40–G41 |
|  | SLP-G | Organic sleep disorders | G47 |
|  | HDG | Headache and migraine | G43–G44, R51 |
| Other / monitoring categories | OTH-G | Other selected G-chapter categories | e.g., G20–G21, G35 |
|  | POIS | Poisoning-related categories | T40, T42, T43 |
|  | OTHER-F | Other F-chapter diagnostic categories | Remaining F codes |
